# ^1^H NMR Metabolomics Study of Spleen from C57BL/6 Mice Exposed to Gamma Radiation

**DOI:** 10.4172/2153-0769.1000165

**Published:** 2016

**Authors:** X Xiao, M Hu, M Liu, JZ Hu

**Affiliations:** 1; 2

**Keywords:** ^1^H NMR metabolomics, Gamma radiation, Spleen, PCA, OPLS, Spectral deconvolution

## Abstract

Due to the potential risk of accidental exposure to gamma radiation, it’s critical to identify the biomarkers of radiation exposed creatures. In the present study, NMR based metabolomics combined with multivariate data analysis to evaluate the metabolites changed in the C57BL/6 mouse spleen after 4 days whole body exposure to 3.0 Gy and 7.8 Gy gamma radiations. Principal component analysis (PCA) and orthogonal projection to latent structures analysis (OPLS) are employed for classification and identification potential biomarkers associated with gamma irradiation. Two different strategies for NMR spectral data reduction (i.e., spectral binning and spectral deconvolution) are combined with normalize to constant sum and unit weight before multivariate data analysis, respectively. The combination of spectral deconvolution and normalization to unit weight is the best way for identifying discriminatory metabolites between the irradiation and control groups. Normalized to the constant sum may achieve some pseudo biomarkers. PCA and OPLS results shown that the exposed groups can be well separated from the control group. Leucine, 2-aminobutyrate, valine, lactate, arginine, glutathione, 2-oxoglutarate, creatine, tyrosine, phenylalanine, π-methylhistidine, taurine, myoinositol, glycerol and uracil are significantly elevated while ADP is decreased significantly. These significantly changed metabolites are associated with multiple metabolic pathways and may be potential biomarkers in the spleen exposed to gamma irradiation.

## Introduction

In our daily life, the potential risk of accidental exposure to ionized radiation is increasing [1]. For example, the nuclear energy landscape is expanding rapidly all over the world, and some of these nuclear energy plants are located in earthquake-prone zones or near seashores [2], like the Fukushima Daiichi nuclear power plant in Japan which suffered major damage from the earthquake and tsunami hit in 2011. Gamma radiation is a major component of ionized radiation from the nuclear accident. Understand the biological impact of gamma radiation to mammal is importance for developing medical counter measures to mitigate the damage from gamma radiation. To achieve this goal, it’s critical to understand thoroughly the biological respond at molecular level, including identify potential biomarkers of radiation exposed creatures for accurate assessment. Gamma radiation disturbances have serious consequences on the whole immune system [3] and the spleen plays important roles in immune system.

Metabolomics is a holistic systems approach capable quantitative measurement metabolic responses of a living system during exposure to external stimuli [4]. ^1^H NMR based metabolomics is a conventional method to explore systematic biochemistry due to almost all metabolites is hydrogen containing molecules [5] and the nature abundance of ^1^H is 99.985%.

As an integrated part of metabolomics, multivariate statistical analysis methods are used for exploring the latent structures embedded within a set of complex data [6]. Generally, there are two pattern recognition methods have widely used in the field of metabolomics, e.g. principal component analysis (PCA) and orthogonal projections to latent structures analysis (OPLS). As an unsupervised pattern recognition method, PCA is the basis of all multivariate data analysis, and the aim is to reduce the dimensionalities of the metadata so that the linear latent variables are orthogonal to each other and keep most information [7]. As an extension of partial least squares (PLS), orthogonal projection to latent structures analysis (OPLS) has received more and more attention since it has been proposed due to its powerful capability in classification and interpretation [8]. In OPLS model, variables in X-matrix are separated into two parts, one is predictive to Y-matrix and the other is orthogonal to Y-matrix, therefore improve the model interpretability of PLS by emphasizing the predictive component [9], and eliminating orthogonal component that is often related to systematic errors associated with, e.g. spectrometer drift, sample storage and processing, etc. Based on OPLS model, a powerful visualization and interpretation tool named S-plot was proposed for multivariate classification model [10], enabling identifying and extracting statistically and significant biomarkers from multivariable data *via* correlation coefficients.

Usually, the pattern recognition methods in the field of NMR based metabolomics are constructed using a reduced variables dataset obtained by binning the original NMR spectra [11]. The method of spectral binning is very expeditious for large scale sample matrix and can be easily automated and pre-processed [12]. However, to identify biologically significant metabolites conventional 2D NMR spectra are needed to assign the signal peaks, a time consuming process for both spectra acquisition and interpretation [13,14].

Recently, mass spectrometry based metabolomics has been successfully utilized for assessing potential biomarkers in urine [15] and plasma [16], meanwhile ^1^H NMR based metabolomics has been utilized for serum [17] from mice exposed to gamma radiation, and interesting results have been obtained. Despite the attractive nature of non-invasive or minimal invasive, these earlier efforts are all reflections of whole system response. Efforts are still needed to assess individual organ or tissue damaged. The reasoning behind is that different genes are active in different kinds of cells in the organism, and the metabolome is also depend on individual, organ and cell type [18]. In this study, the metabolic changed in mouse spleen after whole body exposure to different dosages of gamma irradiation is investigated *via* ^1^H NMR based metabolomics. Specifically, ^1^H NMR spectroscopy was used to detect the hydrophilic metabolites extracted from the excised spleens of the control and exposed mice. Both spectral binning and spectral deconvolution methods are used for generating the data for multivariate data analysis. Two normalization strategies are used, one is the constant sum (i.e., the integration of a metabolite peak/binned data point or peaks related to one metabolite divided the total spectral area) and the other one is unit weight of spleen tissue before extraction. Multivariate data analysis methods (both PCA and OPLS) are used for pattern recognition and identifying a series of metabolites that are statistically and significantly changed as a result of whole body exposure to gamma irradiation in the spleens. Based on these findings, the metabolites pathways that are affected by gamma irradiation are discussed. In addition, the advantages and disadvantages of different pre-process strategies of the NMR spectra and the different approaches of normalization are discussed.

## Materials and Method

### Animal experiments and sample preparation

A total of 17 seven-week-old C57BL/6 female mice were purchased from the Jackson Laboratory (Bar Harbor, ME). After acclimation for one week at the animal facility of Pacific Northwest National Laboratory (PNNL), they were randomly grouped before whole body gamma irradiation using a high activity gamma source (1250 keV ^60^Co). The linear energy transfer (LET) associated with these fields is in the range of 0.2–2 keV/µm. For the whole body gamma radiation, the animals were isolated to the corner of their polymer cages, placed at a minimum of 100 cm from the collimated 6000 Ci ^60^Co source, and then irradiated to the proposed dosage. After irradiation the isolation barrier was removed and animals transferred to PNNL animal facility. The ^60^Co radiation field at the position of mice was measured beforehand using a reference class ionization chamber that was calibrated at the National Institute of Standards and Technology. The resulting absorbed dose rate at approximately 600 mg/cm^2^ depth was 0.83 Gy/min relative to tissue. Groups of mice were exposed to radiation doses of 0 Gy (control, n=8), 3.0 Gy (n=5) and 7.8 Gy (n=4, lethal radiation doses [19]). After 4 days post exposure, mice were sacrificed with 70/30 CO_2_/O_2_ and the spleen from each mouse was excised and cut the same part immediately for each spleen, snap-frozen in liquid nitrogen, then weighted and stored at −80 °C freezer until NMR analysis. All animal work was approved by the Institutional Animal Care and Use Committee (IACUC) at PNNL.

Hydrophilic metabolites were extracted from spleen tissue using a modified Folch method by following the established protocol [20], which was recognized as being able to generate the highest yields under mild extraction conditions [21,22]. It has been generally accepted that about 95% or more of tissue lipids are extracted during the first step [23]. The extraction procedures are briefly described as below:

Step 1: Weight each intact frozen spleen tissue sample about 10 mg. Add 0.25 ml methanol, 0.053 ml deionized water and 0.125 ml chloroform for each tissue sample. All solvents/water used for extraction were placed inside ice bath. Homogenize the mixture while the glass vial was placed inside ice bath using the Tissue Tearor (Model 985-370, BioSpec Products, Inc.). Follow by vortexing for 2 minutes. Step 2: Add 0.125 ml chloroform and 0.125 ml deionized water into the sample then vortex again for 2 minutes. Leave glass vial containing the mixture on ice for 15 minutes, followed by centrifuging at 6,000 rpm for 16 minutes at 4°C. Transfer the lipid and the water soluble layers into glass vials separately with syringes. Finally, the solvents for the water soluble, i.e., the MeOH/H_2_O layer (hydrophilic metabolites) were removed by lyophilizer. And the extracts were stored at −80°C freezer before NMR measurements.

### ^1^H NMR spectroscopy

The hydrophilic metabolites were reconstituted in 500 µl of D_2_O containing 0.05 mM 4, 4-dimethyl-4-silapentane-1-sulfonic acid (DSS) as chemical shift reference and internal concentration standard, and 0.2 % sodium azide as bacteriostatic agent to prevent biodegradation. About 450 µl of the prepared sample was loaded into a standard 5 mm NMR tube (Wilmad, Vineland, NJ, USA). All ^1^H NMR spectra were acquired on a Varian 600 MHz NMR spectrometer equipped with a Z axis-gradient 5mm HCN probe at 20°C. One-dimension ^1^H NMR spectra were acquired from each sample using the standard Varian PRESAT pulse sequence with a single pulse excitation and 1s low power presaturation at the water peak position to suppress the residual water signal. The acquisition time and recycle delay (RD) of a single scan was 3 s and 1 s, respectively, and the spectral width was 7200 Hz. A total of 20 k transients were accumulated, corresponding a total measuring time of about 22 hours for each sample, to ensure that a high quality ^1^H spectrum was obtained with sufficient signal to noise ratio for metabolites with concentration as low as about 0.5 µM or even lower in the NMR tube.

### NMR data processing and multivariate data analysis

All free induction decays (FIDs) were multiplied by an exponential function with a line broadening factor of 0.5 Hz. Prior to Fourier transformation (FT), all FIDs was zero-filled to 128 k data points. Then all ^1^H NMR spectra were phased and baseline corrected manually using the Processor module of Chenomx (NMR suite 8.1, Professional) and referenced to the chemical shift of DSS (CH_3_ peak) at 0 ppm. Two strategies were used to reduce the original spectral data points, i.e., spectral binning and spectral deconvolution. For each approach, two different normalization methods were employed prior to multivariate statistical analysis, i.e., normalization to constant sum [24] and normalization to unit weight of each tissue that was used for extraction [25].

### Spectral binning

NMR-based metabolomics data contain multidimensional metadata points. In particular, the number of data points associated with the spectral dimension is large (i.e., 64 k or more). To analysis large number of spectra by statistics, the method of metadata point reduction such as spectral binning is often used to reduce the number of variables that must be considered [26]. As a conventional data pre-processing method in metabolomics, spectral binning is a rapid and consistent method to produce a reduced set of variables for modeling purpose, where the effect of binning width in a ^1^H spectra of bio-samples to the statistical outcomes was extensively investigated previously [9]. Due to its tremendous advantage of fast automation with short response time, spectral binning has been successfully applied in metabolic profiling in clinical settings to access the disease status of a patient [27]. As a representative bin width, 0.04 ppm is frequently used or recommended for ^1^H spectra owing to it’s a good balance between resolution and the chemical shift influenced by the pH and ionic strength of samples [12]. In the present study, for the hydrophilic extracts of spleen the spectral regions at 0.5–9.0 ppm are segmented into discrete bins with equal width of 0.04 ppm using the Profiler module of Chenomx (NMR suite 8.1, Professional). Spectral regions at δ 0.60–0.66, 1.72–1.80, and 2.88–2.94 (internal standard DSS), 3.28–3.42 (residual methanol) and 4.6–5.15 (residual water), are discarded.

In the present study, we compare two normalization methods for spectral binning data, i.e. relative bin area percent (normalization to constant sum of entire spectrum) and absolute bin areas (normalization to unit weight of spleen tissue before extraction). The relative bin area for each bin data point (i.e., normalization to constant sum) is calculated by dividing each bin area by the total summed bin area in the spectrum. This method is a conventional method when the detailed bio-sample information (i.e., bio-fluids volumes or tissues weight) is not known or ambiguous and credible statistical results can still be achieved as reported previously [28,29]. However, it is not known whether the decrease or increase of the signal intensities of the relative bins is true reflections of the biological pathway modulation. On the other hand, the absolute bin area is calculated by normalizing the bin area to per unit weight of spleen tissue before extraction. As NMR is quantitative, it is expected that the up or down regulate of the bins are true reflections of biological pathway modulations. The normalized NMR spectral bins based on both the absolute and the relative bins are imported into SIMCA (Version 13.0.3, Umetrics, Sweden) for multivariate data analysis (i.e., PCA and OPLS), respectively. One task of the present study is to clarify the advantages or disadvantages of the absolute and the relative bins that has not yet been discussed in literatures so far. Considering every variable of equal importance for statistical analysis, prior to PCA spectral binning data associated with the two normalization strategies discussed above are further mean-centered and unit-variance scaled [30]. PCA is firstly performed to obtain an overview of the data and detect the potential outliers. Subsequently, OPLS is conducted using the auto-scaled data as X-matrix (each row represent a sample, each column represent a variable) and class information as Y-matrix (e.g. 0 for control and 1 for treatment) to find statistically significant variables, i.e., chemical shift integral regions, responsible for the discrimination of the two different classes. The number of principal components obtained from PCA and OPLS analysis is determined by the seven-fold cross-validation. Model quality can be assessed by the parameters R^2^ and Q^2^ that reveal the interpretability of the model and indicate the predictability of the model, respectively. The correlation coefficient plot (i.e., the S-line plot in SIMCA-13) is tailor-made for NMR spectroscopy data based on OPLS model. It visualizes the loading colored according to the absolute value of the correlation coefficient, and can obtain a list of potential variables (spectral bins) that are statistically significant. To obtain the bins that are statistically significant, the cutoff value (Pearson correlation coefficient) depending on the degree of freedom and the discrimination significance (i.e., 95% confidence level, p<0.05) plays a critical role. And the diagnostic tool CV-ANOVA (cross validation analysis of variance) test is further used to evaluate the reliability of the OPLS model (p<0.05).

### Spectral deconvolution

The spectral binning is a powerful and fast method for preprocessing an NMR spectral data set as it can be fully automated. However, spectral binning requires acquiring high quality NMR spectra with each of the spectra having exactly the same residual water peak, baseline and peak shifts for each metabolite that are often very difficult to achieve experimentally [31]. Another even more serious drawback related to spectral binning is that no established technology exists for dealing with overlapping peaks from different metabolites. Therefor a novel method based on spectral deconvolution by using standard spectra on a library of known metabolites has been proposed for mixture analysis, and is defined as “targeted profiling”. The advantages of spectral deconvolution technique are validated against the traditional spectral binning analysis on the basis of sensitivity to water suppression and baseline shift [32]. To avoid the puzzler related to overlapping peaks, in the present study we decide to apply targeted profiling (i.e. the spectral deconvolution). We will compare the results obtained from spectral deconvolution with those obtained from spectral binning method. The method of spectral deconvolution not only assigns spectral peaks with chemical identity but is also able of identifying metabolites with concentrations higher than the detection limits, i.e. 0.3 µM in the NMR tube. By taking advantages of the spectral peak features from resolved peaks of various metabolites, the overlapped signal peaks can be adequately deconvoluted. In this way, the chemical identities associated with the statistically significant metabolites rather than the abstract chemical shift regions are obtained. Similar to the spectral binning data, two normalization strategies (i.e., normalization to constant sum and normalization to unit weight) are used before multivariate data analysis.

The strategy of spectral deconvolution offers us the advantage of determining the estimated absolute concentration of each metabolite in the tissue. In general, achieve the assignments of spectral peaks are cross validated using a suit of conventional 2D NMR spectra such as ^1^H-^1^H correlation spectroscopy (COSY), ^1^H-^1^H total correlation spectroscopy (TCOSY), ^1^H J-resolved spectroscopy (JRES), ^1^H-^13^C heteronuclear single quantum correlation spectroscopy (HSQC) and ^1^H-^13^C heteronuclear multiple bond correlation spectroscopy (HMBC) as previously reported [33,34]. In the present study the procedures described below. First, the NMR spectrum is deconvoluted and the concentration of each metabolite is determined by the well-established method provided by Chenomx (NMR suite 8.1, professional) using the known concentration of DSS as internal standard. The spectral deconvolution is performed on the Profiler module of Chenomx with database containing more than 330 common metabolites associated with mammals and bacteria. Secondly, the concentration of each metabolite is further normalized to per milligram of spleen tissue before extraction. In parallel, a second data set with normalization to constant sum is obtained using a simple calculation, i.e., dividing the concentration of each metabolite by the sum of the concentrations of all metabolites. Multivariate data analysis (e.g. PCA and OPLS) are carried out in exactly the same way as described earlier for spectral binning data.

## Results and Discussion

### NMR spectra of metabolites from spleen tissue extracts

**Figure 1:**
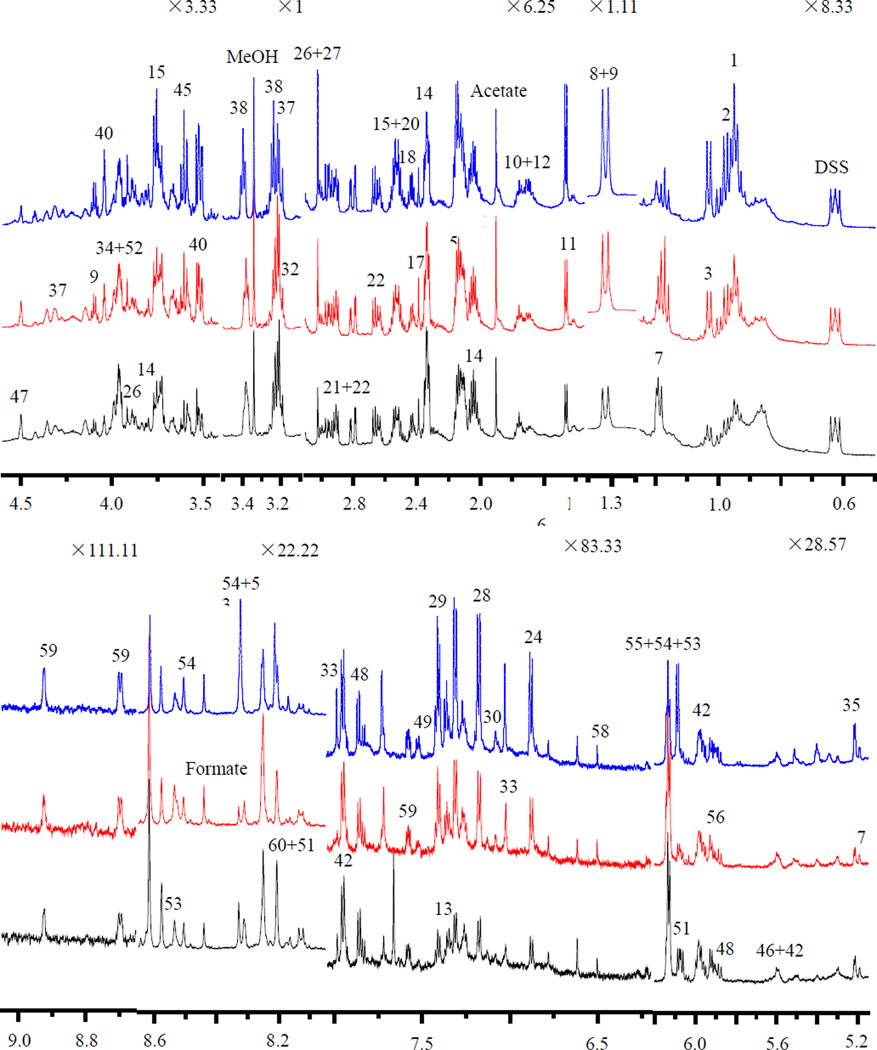
600 MHz liquid state 1H NMR metabolites spectra of the hydrophilic extracts of spleens excised from the control and the radiation exposure mice. The peak intensities were normalized to per unit weight of spleen before extraction. In this plot, spectral regions between different chemical shifts are vertically expanded by different times to highlight the peaks of varied spectral intensities. Black: control, red: 3 Gy, blue: 7.8 Gy.

Examples of typical ^1^H NMR spectra of hydrophilic extracts obtained from a control mouse, mice exposed to 3 Gy and 7.8 Gy whole body gamma irradiation are shown in Figure 1. In the plot, the peak intensities are normalized to unit weight of spleen tissue before extraction, so the peak intensities in the spectra of radiation group and those of the control group can be directly compared visually.

A total of 61 metabolites are assigned with good confidence according to the literature reports [35,36] and the database of Chenomx. These assignments are further confirmed by 2D NMR spectra (e.g. COSY and JRES, Figures S2 and S3). The peaks assignment and basic statistical parameters associated with each detectable metabolite from spectral deconvolution are listed in Table 1, including the mean concentrations and standard deviation. A variety of amino acids, carbohydrates, glycolysis and citrate acid cycle intermediates are detected. And other metabolites include choline metabolites, ethanolamine metabolites, organic bases were observed. Visual inspection of the ^1^H NMR spectra indicates apparent metabolism alterations induced by gamma irradiation. For example, the radiation exposure mice have higher level of leucine, 2-aminobutyrate, threonine, valine, lactate, alanine, arginine, myo-inositol, malate, taurine, 2-oxoglutarate, glycerol and glutathione in the radiation groups (3Gy and 7.8Gy) in Figure 1, while the bottom trace spectrum (control) shows evidently higher level of isocitrate, o-phosphoethanolamine, betaine, UDP-glucose, ascorbate and inosine. To discern statistically and significantly changed metabolites, PCA and OPLS statistical analyses on the entire spectral sets containing a total of 17 mice from both control and gamma-irradiated groups are performed.

### Statistical results based on spectral binning

**Figure 2:**
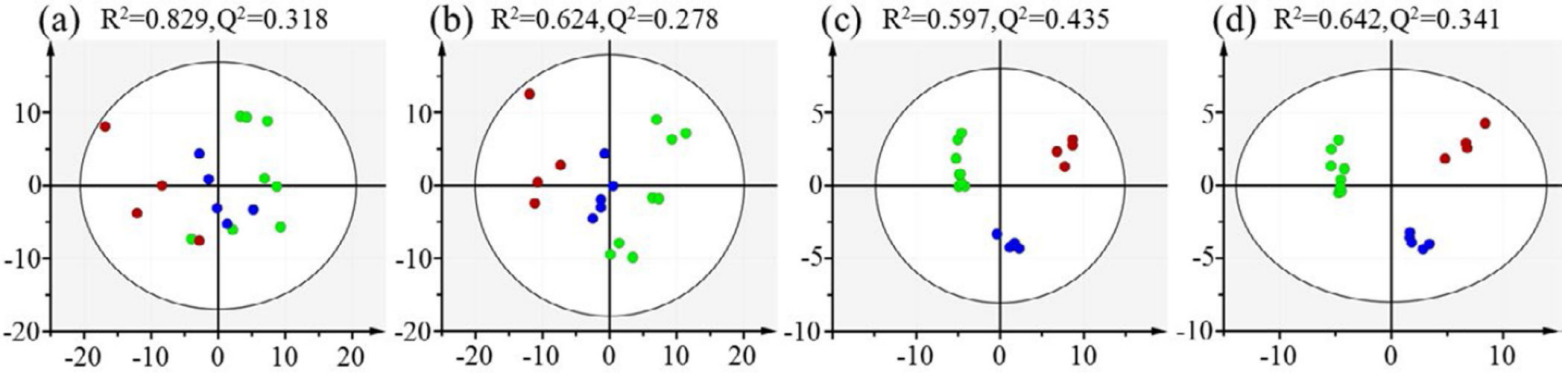
PCA scores plots of spleen tissue extracts from the control (green dots), 3 Gy (blue dots) and 7.8 Gy (red dots) irradiation groups: (a) binning results of ^1^H NMR spectra with normalization to unit weight, (b) binning results of ^1^H NMR spectra with normalization to constant sum, (c) metabolites concentrations obtained by spectral deconvolution and normalization to unit weight, (d) metabolites concentrations obtained by spectral deconvolution and normalization to constant sum.

**Figure 3:**
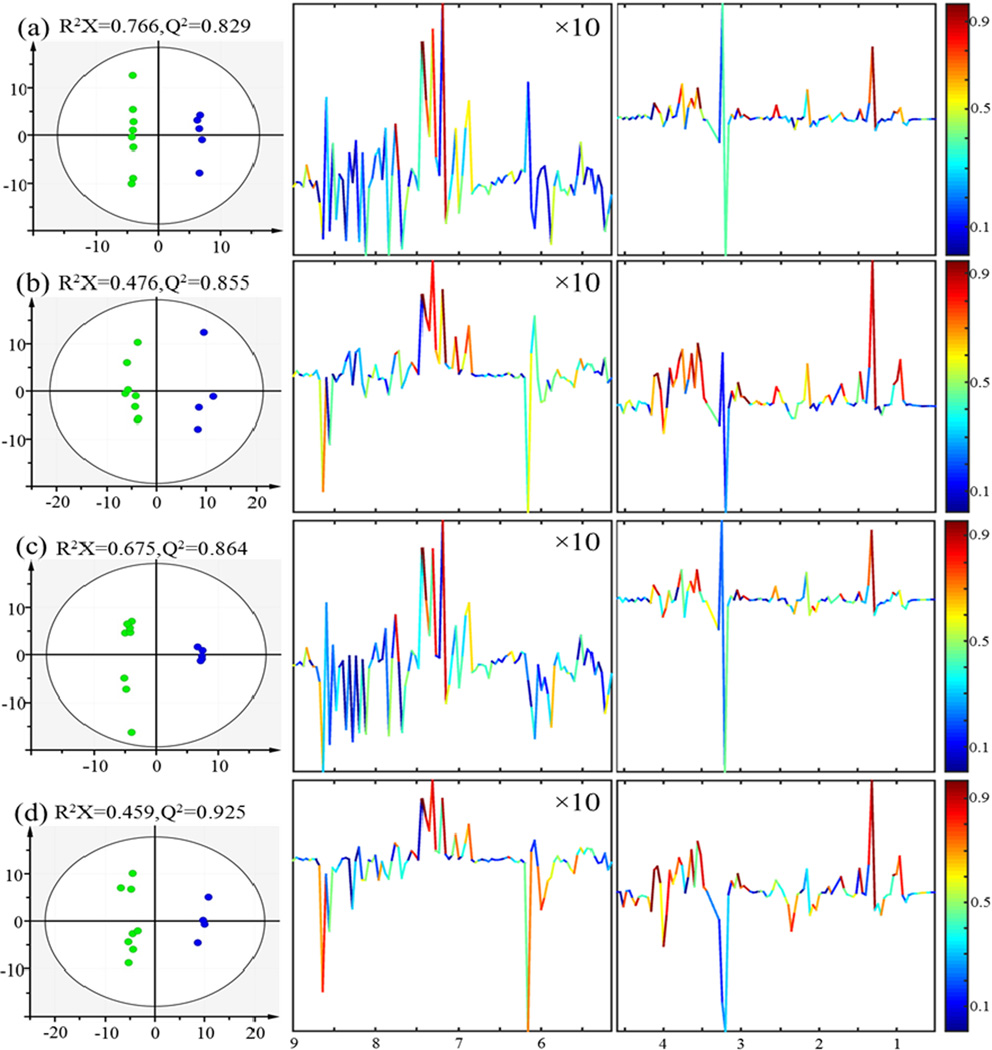
OPLS scores (left) and coefficients-coded loading plot (right) of the model discriminating the control (*green dots*) and the radiation (*blue dots*) groups. (a) Data derived from binning results of control and 3 Gy and normalization to unit weight, (b) Data derived from binning results of control and 7.8 Gy and normalization to unit weight, (c) Data derived from binning results of control and 3 Gy and normalization to constant sum, (d) Data derived from binning results of control and 7.8 Gy and normalization to constant sum. CV-ANOVA results gave p values of 0.095, 0.046, 0.02 and 0.005 for models (a), (b), (c) and (d), respectively.

Data sets consisting of relative bin area (normalization to constant sum) and absolute bin area (normalization to unit weight) are subjected to multivariate data analysis (e.g. PCA and OPLS). The PCA scores plot shown that the three groups (control, 3 Gy and 7.8 Gy) are clearly separated without any outliers based on relative bin area (Figure 2b). In order to maximize the correlation between X-matrix (the integral area of spectral bins) and Y-matrix (the class information) as well as the variation in X-matrix, OPLS is performed to evaluate and identify discriminatory variables responsible for separation between different groups. The variables shown significance difference between control and treatment groups are extracted from the correlation coefficients-coded loadings plot of the OPLS model constructed. The parameters R^2^X and Q^2^ shown good quality of the generated OPLS model, and CV-ANOVA results further confirm the model validity (p<0.05) (Figure 3). Obviously, spectral binning is a rapid and easily automated data reduction strategy, especially for large scale of samples. It is applicable in evaluating changes between two groups where decisions have to be made within a shortly time. And the results of statistical perform on the relative bin area percent is better than the absolute bin, primarily because the integral area is prone to the influence by the inconsistent baseline shifts between samples. In addition, there is an inherent drawback related to spectral binning. As shown in S-plot (Figure 3), the significant variables are chemical shift values (the small chemical shift regions) rather than the chemical identities of specific metabolites. The corresponding “bins” that cannot be assigned to the specific metabolites are from bins related to spectral peaks with heavy overlap from different metabolites.

For example, in Figure 3, variables with δ around 1.3 and 4.1 are considered significant because the absolute values of their corresponding correlation coefficients are large than the Pearson correlation coefficient, and they can be easily identified as lactate due to no overlap with other high intensity peaks. However, in spectral regions such as variables with δ 2.4–2.5 (glutathione, isocitrate and β-alanine with metabolites key 15, 19 and 20, respectively, in Table 1), and variables with δ 4.2–4.3 (threonine and malate with metabolites key 8 and 16, respectively, in Table 1), many metabolites contribute to the same peak. Obviously, these variables cannot be assigned to any individual metabolite due to severe metabolites peaks overlapped.

### Statistical results based on spectral deconvolution

Considering the inherent disadvantage related to the spectral binning, spectral deconvolution is further to identify specific metabolites responsible for separating the gamma-radiation exposed groups from the control group. Multivariate data analysis methods (e.g. PCA and OPLS) are performed directly on the absolute concentrations of metabolites that are normalized to per milligram of tissue before extraction and relative concentration percent that normalized to constant sum of all metabolites concentration, respectively. Multivariate data analyses are carried out in exactly the same way as mentioned before in spectral binning section. PCA scores plots (Figures 2c and 2d) has shown clear classification of the control and treatment groups based on both absolute and relative concentrations.

Since no outliers are detected by PCA, all 17 samples are kept for OPLS model analysis. In the PCA scores plot (Figure 2), the control, 3 Gy and 7.8 Gy groups are better separated than those of spectral binning results. The OPLS model statistical analysis parameters, i.e. the R^2^X explains the variance in X-matrix and Q^2^ explains the predictive performance, both also show better statistical performance than the corresponding spectral binning results (Table S6). Moreover, the p-values (<0.05) from CV-ANOVA show that the OPLS models are valid using either multivariate data normalization strategy. Based on these statistical parameters, we conclude that spectral deconvolution is better than spectral binning for dealing with overlapping spectral peaks and for identifying the chemical identities of discriminatory metabolites between the control and the treatment groups in addition to better separating them.

The method of normalization to estimated absolute tissue concentration is the common method in the field of biology and also in metabolomics applications [37,38]. The use of relative concentration (i.e., normalization to constant sum) for multivariate data analysis is the conventional way when the weight of tissue before extraction is not known [39]. However, the relative concentration is prone to take the risk of achieving pseudo biomarkers. We will emphasize this pitfall late by using the known metabolite concentrations given in Table 1.

With normalization to constant sum, there are 16 metabolites found statistically and significantly different between the 3 Gy exposed and the control group (Table S3) while there are 21 metabolites found statistically different when the method of normalization to unit weight is used on the same data set. Although there are 14 metabolites found statistically significant regardless of the normalization method used, there are metabolites with no statistical importance associated with one method become statistically important with the other method. For example, taurine, a high concentration metabolite (with metabolite key of 38 in Table 1) is of high statistical significance when normalization to unit tissue weight is used but shows no statistical importance when normalization to constant sum is used. The same finding applies to 2-oxoglutarate, glutathione, glycerol, glycine, malate, and π-methylhistidine with metabolite concentration across a wide range. In contrast, o-phosphoethanolamine and sn-glycero-3-phosphocholine are not statistically significant with normalization to unit weight but become statistically important with normalization to constant sum. Similar observations are found for the 7.8 Gy data shown in Table S4, where there are 15 statistically important metabolites with normalization to constant sum while there are 22 metabolites with normalization to unit tissue weight. Considering normalization to unit weight tissue mass is the gold standard for traditional biology, using the method of normalization to constant sum should be very careful due to the following potential shortcomings. (i) Metabolites that are statistically important by natural may be overlooked or missing; (ii) There may be pseudo biomarkers mistaken as up or down regulated in biological pathways. Therefore, in the following we will use the method of normalization to unit weight of tissue mass to discuss the statistically important metabolites and relate them to the biological pathways. Also to simplify the discussion, only the metabolites that are statistically important to both the 3.0 Gy and 7.8 Gy groups when compared with the controls are discussed below.

It can conclude from the Table 1 and Table 2 based on the estimated absolute metabolite concentrations in tissues that compared with the control group, in the radiation groups the concentrations of ADP is decreased statistically and significantly, while the concentrations of leucine, 2-aminobutyrate, valine, lactate, arginine, glutathione, 2-oxoglutarate, creatine, tyrosine, phenylalanine, π-methylhistidine, taurine, myo-inositol, glycerol and uracil are increased statistically and significantly. All these statistically significant changed metabolites can be considered as potential biomarkers of metabolism disturbance induced by gamma radiation in spleen.

## Discussion

It is known that gamma irradiation damages DNA *via* double strand break, induces oxidative stress [40] and increases protein turnover [41] (Table S6). The genes in DNA encode protein molecules that are the "workhorses" of all cells, carrying out all the functions necessary for life. Such as almost all enzymes, including those that metabolize nutrients and synthesize new cellular constituents, are proteins [42]. The metabolites are the end or intermediate products of cellular regulatory processes and most of biochemistry reaction catalyzed by enzyme [43]. So the gamma radiation damaged the DNA double strand and induced the metabolic disturbed.

In this study, it is shown that the 3 Gy and 7.8 Gy irradiation groups are separated from the control group based on PCA analysis. Sixteen metabolites have been found statistically different between the control and the treatment groups. Up-regulated metabolites included leucine, 2-aminobutyrate, valine, lactate, arginine, glutathione, 2-oxoglutarate, creatine, tyrosine, phenylalanine, π-methylhistidine, taurine, myo-inositol, glycerol and uracil. Down regulated metabolite is ADP.

Clearly, many of the statistically significant metabolites in spleen arising from gamma-radiation damage belong to the amino acid family, including leucine, valine, arginine, tyrosine, phenylalanine, π-methylhistidine and taurine, etc. The up regulation of leucine, valine, arginine, tyrosine, and phenylalanine have been previously attributed to the result of DNA double strand break and double strand break induced mutation in codon [44], and the increased protein turnover will release of these amino acids [45]. The carbon skeletons of leucine and tyrosine are degraded to produce acetyl-CoA that enters into the critic acid cycle. It has been reported that leucine is capable of protecting animals against oxidative stress [46]. Tyrosine can be used as an effective radio protector against protein damage [47]. The unmodified tyrosine could protect DNA against radiation induced strand breaks [48]. Phenylalanine is a precursor for tyrosine that yields fumarate into the citric acid cycle by a specific organic catalyst called phenylalanine hydroxylase. A genetic defect in phenylalanine hydroxylase has been reported as the most common cause of elevated levels of phenylalanine [49]. The carbon skeletons of valine can be combined with other amino acids to yield succinyl-CoA, an intermediate of the citric acid cycle. The increase of valine level reflects radiation induced valine-rich protein breakdown and inactivate some enzymes, regulating pathway that produce succinyl-CoA [50]. Arginine plays an important role in cell division, removing ammonia from body and immune function; the radiation can induced immune dysfunction [51]. The carbon skeletons of arginine enter the citric acid cycle as 2-oxoglutarate. Taurine is an organic acid widely distributed in animal tissues and the regulation of oxidative stress [52]. Glutathione is the major endogenous antioxidant in animal cells, and can be used in metabolic and biochemical reactions such as DNA synthesis and repair [53]. Creatine is an important substrate of creatine kinase that constitutes a complex cellular energy buffer. The administration of creatine stabilizes the mitochondrial creatine kinase and prohibits opening of the mitochondrial transition pores [54]. It has been reported that the administration of creatine can protect radiation exposed mice from increasing in biochemical indices of oxidative stress [55]. Therefore, creatine has been suggested as a new therapeutic drug for treating gamma radiation. Oxidative stress can induce tryptophan metabolism disturbance and the increased tryptophan level could attenuate the oxidative stress of the spleen [56].

Other metabolites that show statistically and significant differences between the control and the gamma-radiation exposed groups are related to the energy metabolism of the citrate acid cycle, including 2-aminobutyrate, 2-oxoglutarate and lactate with all of them found up-regulated. The increased 2-aminobutyrate indicates that the cells suffer oxidative stress [57]. 2-oxoglutarate plays a critical role in DNA double strand break synthesis that damaged by the irradiation. A kind of DNA repair enzyme is a 2-oxoglutarate dependent Fe^2+^ binding dioxygenase that removes methyl lesions from DNA. Formation of a fully folded and the catalytically competent enzyme only occurs when both 2-oxoglutarate and Fe^2+^ are bound [58]. Gamma radiation induces energy metabolism disturbance, resulting in high levels of lactate production. Lactate is then transferred from these glycolytic fibroblasts to adjacent cells and be used as fuel for oxidative mitochondrial metabolism [59].

Oxidative stress causes the inactivation of several key enzymes so that the inhibition of glycolysis and beta-oxidation leads to the metabolism towards glycerol production [60]. Irradiation induces damage to DNA *via* double-strand breaks, oxidative base lesions in DNA are mainly repaired by base excision [61]. Uracil is the main substrate of uracil-DNA glycosylases. The increased uracil is the result of DNA repair [61]. Myo-inositol is a versatile compound and plays an important role in generating diversified derivatives upon phosphorylation. Phosphatidylinositol form one such group of myo-inositol derivatives that act both as membrane structural lipid molecules and as signals. The increased myo-inositol indicates synthetized cell membrane that is damaged by irradiation. The energy metabolism disturbance and the DNA damage repair need more ATP, resulting in a decreased ADP level [62].

## Conclusion

**Figure 4:**
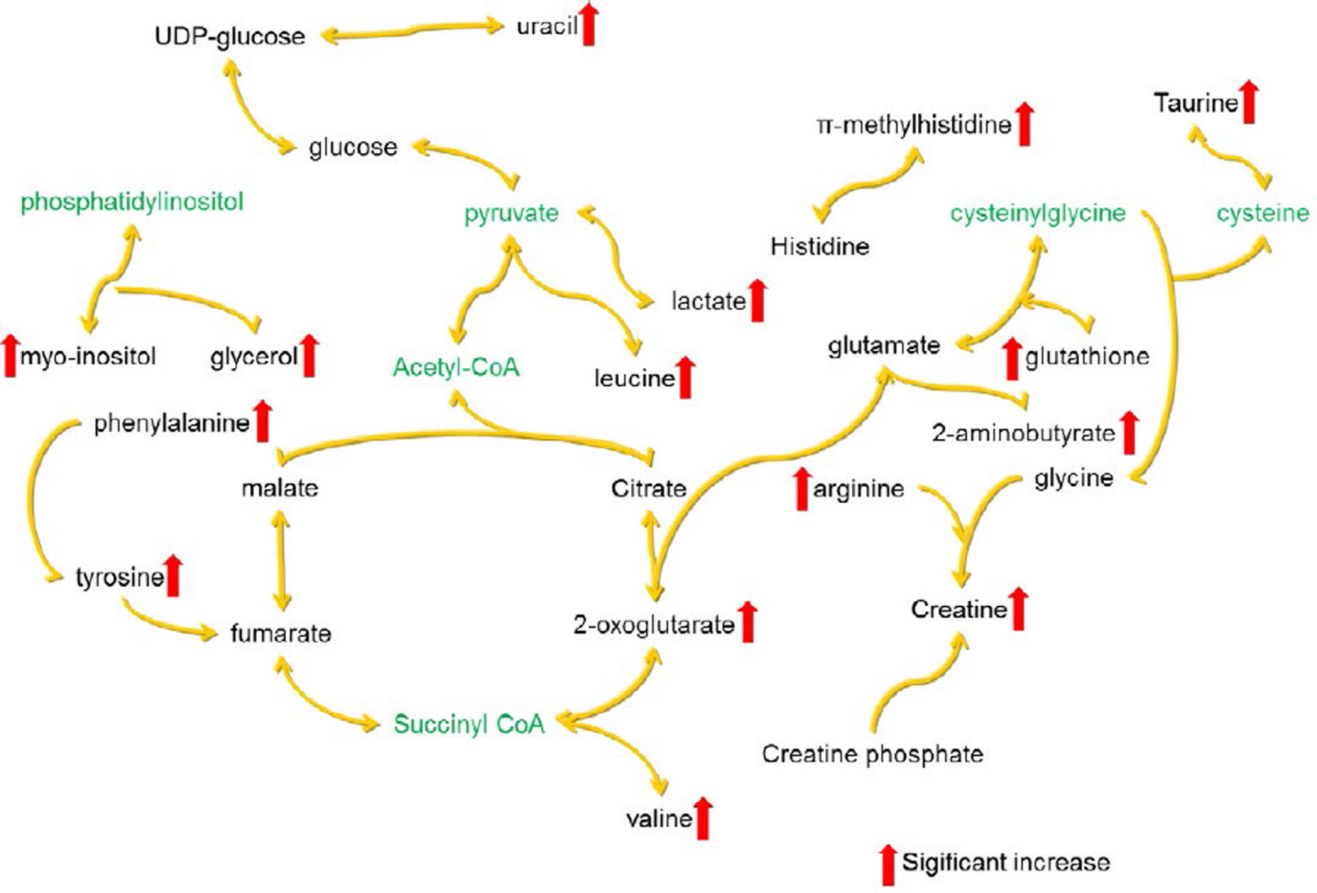
Proposed metabolic pathway networks associated with the significantly altered metabolites after exposed to gamma radiation based on the findings from this work and the diverse metabolic fates depicted in the small molecule pathway database (SMPDB) (http://www.smpdb.ca/). Metabolites colored green are not detected.

We have shown that the combined application of ^1^H NMR metabolomics and multivariate data analysis (e.g. PCA and OPLS) is a powerful tool for exploring gamma irradiation induced metabolites changed in mouse spleen (Figure 4). Both PCA and OPLS shown that the groups exposed to whole body 3.0 and 7.8 Gy radiation at 4 days post exposure are well separated from the control group. A total of 61 metabolites with estimated absolute concentration in spleen tissues ranged from 20 µM to 28.26 mM are identified in the hydrophilic extracts of spleen. Various data pre-process methods are investigated, including spectral analysis involving spectral binning and spectral deconvolution, and normalization methods involving normalize to constant sum or normalize to unit weight. It is found that spectral deconvolution offers better statistical results than spectral binning for identifying the potential biomarkers in mouse exposed to gamma-radiation. While the method of normalization to tissue weight (i.e., the estimated absolute concentration) generate more metabolites that are statistically important than those the constant sum. Normalization to constant sum is also demonstrated at the risk of achieving pseudo biomarkers that could be mistaken as up or down regulated metabolites in biological pathway analysis. Using the combination of spectral deconvolution and normalization to unit tissue weight, it is found that gamma radiation induced metabolic changes in mouse spleen tissue, resulting in statistically and significantly up regulated leucine, 2-aminobutyrate, valine, lactate, arginine, glutathione, 2-oxoglutarate, creatine, tyrosine, phenylalanine, π-methylhistidine, taurine, myo-inositol, glycerol and uracil, and statistically and significantly down regulated ADP. These statistically significant changed metabolites may be potential biomarkers for gamma radiation creature in spleen.

## Supplementary Material

Suppl data

## Figures and Tables

**Table 1:** Hydrophilic metabolites peak assignments and concentrations

Key	Metabolites	δ ^1^H (ppm) and multiplicity*	Concentration (µM/mg)			Estimates of absolute concentrations in tissue (mM)		
			Mean ± SD			Mean ± SD		
			Control	3 Gy	7.8 Gy	Control	3 Gy	7.8 Gy
1	Leucine	0.94 (t), 0.99 (d), 1.27 (m), 1.46 (m), 1.95 (m), 3.64 (d)	1.83 ± 0.30	2.78 ± 0.22	4.44 ± 1.02	0.96 ± 0.16	1.46 ± 0.12	2.34 ±0.54
2	2-Aminobutyrate	0.96 (t), 1.91 (m), 3.68 (t)	1.48 ± 0.06	2.23 ± 0.20	2.37 ± 0.45	0.78 ± 0.03	1.17 ± 0.11	1.25 ± 0.24
3	Valine	0.97 (d), 1.02 (d), 2.27 (m), 3.6 (d)	1.37 ± 0.17	2.38 ± 0.20	4.21 ± 0.71	0.72 ± 0.09	1.25 ± 0.11	2.21 ± 0.37
4	Isobutyrate	1.05 (d), 2.38 (m)	0.22 ± 0.04	0.17 ± 0.01	0.22 ± 0.02	0.12 ± 0.02	0.09 ± 0.01	0.12 ± 0.01
5	3-Hydroxyisobutyrate	1.08 (d), 2.48 (m), 3.53 (m), 3.69 (m)	0.27 ± 0.05	0.19 ± 0.02	0.31 ± 0.05	0.14 ± 0.03	0.10 ± 0.01	0.16 ± 0.03
6	3-Hydroxybutyrate	1.2 (d), 2.3 (dd), 2.39 (dd), 4.14 (dd)	2.60 ± 0.32	2.66 ± 0.32	1.41 ± 0.11	1.37 ± 0.17	1.40 ± 0.17	0.74 ± 0.06
7	Fucose	1.2 (m), 3.4 (dd), 3.63 (dd), 3.8 (m), 4 (m), 4.19 (m), 4.55 (d), 5.22 (m)	1.20 ± 0.17	1.46 ± 0.24	1.68 ± 0.43	0.63 ± 0.09	0.77 ± 0.13	0.88 ± 0.23
8	Threonine	1.33 (d), 3.58 (d), 4.26 (m)	4.69 ± 0.51	7.05 ± 0.91	6.97 ± 0.97	2.47 ± 0.27	3.71 ± 0.48	3.67 ± 0.51
9	Lactate	1.33 (d), 4.11(q)	10.89 ± 1.24	18.7 ± 1.97	24.51 ± 1.57	5.73 ± 0.65	9.84 ± 1.04	12.9 ± 0.83
10	Lysine	1.43 (m), 1.51 (m), 1.72 (m), 1.89 (m), 1.91 (m), 3.03 (t),3.75 (t),	1.13 ± 0.20	1.54 ± 0.08	1.61 ± 0.19	0.59 ± 0.10	0.81 ± 0.04	0.85 ± 0.10
11	Alanine	1.48 (d), 3.78 (q)	3.55 ± 0.52	4.66 ± 0.76	7.06 ± 1.15	1.87 ± 0.27	2.45 ± 0.4	3.71 ± 0.61
12	Arginine	1.65 (m), 1.74 (m), 1.9 (m), 1.92 (m), 3.23 (t), 3.77 (t)	2.04 ± 0.21	3.04 ± 0.40	3.58 ± 0.28	1.07 ± 0.11	1.60 ± 0.21	1.88 ± 0.15
13	γ-Glutamylphenylalanine	1.97 (m),2.34 (m),2.89 (dd),3.21 (dd),3.57 (t),4.46 (m),7.27 (t),7.35 (t),7.91 (d)	0.23 ± 0.02	0.25 ± 0.03	0.33 ± 0.03	0.12 ± 0.01	0.13 ± 0.01	0.17 ± 0.01
14	Glutamate	2.05 (m), 2.14 (m), 2.34 (m), 2.37 (m), 3.76 (dd)	21.34 ± 3.37	21.65 ± 1.30	18.44 ± 1.52	11.23 ± 1.77	11.4 ± 0.69	9.71 ± 0.80
15	Glutathione	2.16 (m), 2.18 (m), 2.52 (m), 2.57 (m), 2.95 (dd), 2.98 (dd), 3.74 (d), 3.77 (m), 4.58 (m)	1.92 ± 0.15	2.65 ± 0.36	3.27 ± 0.54	1.01 ± 0.08	1.40 ± 0.19	1.72 ± 0.29
16	Malate	2.36 (dd), 2.67 (dd), 4.3 (m)	2.18 ± 0.14	2.79 ± 0.27	2.67 ± 0.44	1.14 ± 0.07	1.47 ± 0.14	1.40 ± 0.23
17	Succinate	2.39 (s)	1.13 ± 0.28	1.10 ± 0.09	0.91 ± 0.22	0.60 ± 0.15	0.58 ± 0.05	0.48 ± 0.12
18	2-Oxoglutarate	2.44 (t), 2.99 (t)	1.53 ± 0.11	2.06 ± 0.24	2.83 ± 0.38	0.81 ± 0.06	1.08 ± 0.12	1.49 ± 0.20
19	Isocitrate	2.49 (m), 2.57 (m), 2.99 (m), 4.06 (d)	3.02 ± 0.53	2.83 ± 0.25	3.98 ± 0.38	1.59 ± 0.28	1.49 ± 0.13	2.10 ± 0.20
20	β-Alanine	2.53 (t), 3.20 (t)	1.73 ± 0.18	2.23 ± 0.61	2.41 ± 0.13	0.91 ± 0.09	1.17 ± 0.32	1.27 ± 0.07
21	Citrate	2.54 (d), 2.68 (d)	0.19 ± 0.08	0.28 ± 0.04	0.28 ± 0.04	0.10 ± 0.04	0.15 ± 0.02	0.15 ± 0.02
22	Aspartate	2.67 (dd), 2.82 (dd), 3.9 (dd)	10.22 ± 1.49	10.77 ± 1.17	11.16 ± 1.25	5.38 ± 0.78	5.67 ± 0.61	5.87 ± 0.66
23	Trimethylamine	2.88 (s)	0.15 ± 0.03	0.15 ± 0.02	0.15 ± 0.01	0.08 ± 0.02	0.08 ± 0.01	0.08 ± 0.01
24	Tyramine	2.92 (t), 3.23 (t), 6.9 (m), 7.2 (m)	0.05 ± 0.01	0.07 ± 0.01	0.20 ± 0.06	0.02 ± 0.01	0.03 ± 0	0.11 ± 0.03
25	Creatine phosphate	3.01 (s), 3.94 (s)	0.57 ± 0.11	0.61 ± 0.07	0.84 ± 0.06	0.30 ± 0.06	0.32 ± 0.04	0.44 ± 0.03
26	Creatine	3.04 (s), 3.91 (s)	0.76 ± 0.12	1.67 ± 0.17	1.81 ± 0.15	0.40 ± 0.06	0.88 ± 0.09	0.95 ± 0.08
27	Creatinine	3.04 (s), 3.98 (s)	0.63 ± 0.11	0.64 ± 0.05	1.25 ± 0.08	0.33 ± 0.06	0.34 ± 0.03	0.66 ± 0.04
28	Tyrosine	3.06 (dd), 3.2 (dd), 3.93 (dd), 6.9 (m), 7.2 (m)	0.35 ± 0.04	0.65 ± 0.11	1.04 ± 0.10	0.18 ± 0.02	0.34 ± 0.06	0.54 ± 0.05
29	Phenylalanine	3.11 (dd), 3.27 (dd), 3.99 (dd), 7.33 (m), 7.38 (m), 7.43 (m)	0.41 ± 0.08	0.74 ± 0.11	1.36 ± 0.33	0.22 ± 0.04	0.39 ± 0.06	0.72 ± 0.18
30	Histidine	3.14 (dd), 3.24 (dd), 3.98 (dd), 7.06 (s), 7.79 (s)	0.21 ± 0.05	0.17 ± 0.04	0.27 ± 0.02	0.11 ± 0.02	0.09 ± 0.02	0.14 ± 0.01
31	Ethanolamine	3.14 (m), 3.82 (m)	2.54 ± 0.41	2.31 ± 0.42	2.65 ± 0.58	1.34 ± 0.21	1.21 ± 0.22	1.39 ± 0.31
32	Choline	3.21 (s), 3.52 (m), 4.07 (m)	2.18 ± 0.39	1.35 ± 0.19	2.43 ± 0.27	1.15 ± 0.21	0.71 ± 0.10	1.28 ± 0.14
33	π-Methylhistidine	3.22 (dd), 3.3 (dd), 3.73 (s), 3.95 (dd), 6.8 (s), 8 (s)	0.18 ± 0.02	0.29 ± 0.08	0.42 ± 0.09	0.09 ± 0.01	0.15 ± 0.04	0.22 ± 0.05
34	O-Phosphoethanolamine	3.22 (m), 3.98 (m)	18.22 ± 1.18	15.93 ± 1.33	13.69 ± 1.38	9.59 ± 0.62	8.39 ± 0.70	7.21 ± 0.73
35	Glucose	3.23 (m), 3.4 (m), 3.5 (m), 3.53 (dd), 3.7 (dd), 3.72 (dd),3.78 (m), 3.83 (m), 3.84 (m), 3.94 (dd), 4.65 (d), 5.23 (d)	1.49 ± 0.31	1.49 ± 0.32	2.84 ± 0.80	0.78 ± 0.17	0.79 ± 0.17	1.50 ± 0.42
36	Trimethylamine N-oxide	3.23 (s)	0.95 ± 0.15	0.74 ± 0.17	0.98 ± 0.15	0.50 ± 0.08	0.39 ± 0.09	0.51 ± 0.08
37	sn-Glycero-3- phosphocholine	3.23 (s), 3.6 (dd), 3.67 (m), 3.68 (dd), 3.86 (m), 3.92 (m),3.95 (m), 4.32 (m)	5.49 ± 0.73	7.41 ± 0.98	7.71 ± 1.38	2.89 ± 0.38	3.90 ± 0.52	4.06 ± 0.72
38	Taurine	3.26 (t), 3.43 (t)	39.14 ± 1.12	44.86 ± 1.26	53.7 ± 4.46	20.6 ± 0.59	23.61 ± 0.66	28.26 ± 2.35
39	Betaine	3.27 (s), 3.91 (s)	1.71 ± 0.38	1.04 ± 0.37	1.07 ± 0.22	0.90 ± 0.20	0.55 ± 0.19	0.56 ± 0.12
40	myo-Inositol	3.28 (t), 3.53 (dd), 3.62 (t), 4.06 (m)	7.03 ± 0.69	12.3 ± 1.39	18.98 ± 3.24	3.70 ± 0.36	6.48 ± 0.73	9.99 ± 1.70
41	Tryptophan	3.3 (dd), 3.48 (dd), 4.05 (dd), 7.2 (dd), 7.29 (dd), 7.32 (s),7.54 (d), 7.74 (d),	0.16 ± 0.03	0.28 ± 0.02	0.33 ± 0.06	0.08 ± 0.02	0.15 ± 0.01	0.17 ± 0.03
42	UDP-glucose	3.51 (dd), 3.58 (m), 3.78 (dd), 4.13 (d), 4.17 (m), 4.23 (m), 4.27 (m), 4.36 (m), 5.61 (dd), 5.97 (d), 5.99 (d),7.97 (d)	1.08 ± 0.12	0.86 ± 0.01	0.78 ± 0.09	0.57 ± 0.06	0.45 ± 0.01	0.41 ± 0.05
43	UDP-glucuronate	3.51 (dd), 3.58 (m), 3.78 (dd), 4.13 (d),4.17 (m),4.23 (m), 4.27 (m), 4.36 (m), 5.61 (dd), 5.97 (d), 5.99 (d),7.97 (d)	0.34 ± 0.02	0.29 ± 0.06	0.26 ± 0.03	0.18 ± 0.01	0.15 ± 0.03	0.14 ± 0.02
44	Glycerol	3.56 (dd), 3.65 (dd), 3.78 (m)	1.81 ± 0.18	2.58 ± 0.23	3.52 ± 0.41	0.95 ± 0.10	1.36 ± 0.12	1.85 ± 0.21
45	Glycine	3.56 (s)	3.76 ± 0.85	5.99 ± 0.88	4.25 ± 0.97	1.98 ± 0.45	3.16 ± 0.46	2.23 ± 0.51
46	UDP-galactose	3.72 (dd), 3.75 (dd), 3.81 (m), 3.91 (dd), 4 (d), 4.16 (m),4.19 (m), 4.24 (m), 4.28 (m), 4.37 (m), 5.63 (dd), 5.98 (m), 7.95 (d)	0.27 ± 0.04	0.29 ± 0.06	0.29 ± 0.03	0.14 ± 0.02	0.15 ± 0.03	0.15 ± 0.02
47	Ascorbate	3.73 (m),4.00 (t),4.50 (d)	9.00 ± 1.13	7.98 ± 0.71	6.32 ± 2.49	4.74 ± 0.59	4.20 ± 0.38	3.33 ± 1.31
48	Uridine	3.8 (dd), 3.89 (dd), 4.13 (m), 4.23 (t), 4.36 (t), 5.9 (d),5.92 (d), 7.88 (d),	0.57 ± 0.17	0.70 ± 0.11	0.56 ± 0.05	0.30 ± 0.09	0.37 ± 0.06	0.29 ± 0.02
49	Cytidine	3.81 (dd), 3.92 (dd), 4.12 (m), 4.19 (t), 4.3 (m), 5.89 (d),6.06 (d), 7.81(d)	0.33 ± 0.07	0.26 ± 0.03	0.26 ± 0.04	0.17 ± 0.04	0.14 ± 0.02	0.14 ± 0.02
50	Adenosine	3.83 (dd), 3.91 (dd), 4.29 (m), 4.42 (dd), 4.79 (dd), 6.05 (d), 8.24 (s), 8.34 (s)	0.75 ± 0.18	0.67 ± 0.15	0.42 ± 0.07	0.39 ± 0.09	0.36 ± 0.08	0.22 ± 0.04
51	Inosine	3.84 (dd), 3.92 (dd), 4.27 (m), 4.44 (m), 4.76 (t), 6.11 (d),8.23 (s), 8.35 (s)	0.71 ± 0.39	0.27 ± 0.08	0.31 ± 0.21	0.37 ± 0.20	0.14 ± 0.04	0.16 ± 0.11
52	Serine	3.84 (dd), 3.95 (dd), 3.99 (dd)	6.75 ± 1.23	7.21 ± 0.65	8.96 ± 0.41	3.55 ± 0.65	3.80 ± 0.34	4.72 ± 0.22
53	AMP	4.01 (m), 4.36 (m), 4.49 (m), 4.78 (dd), 6.11 (d), 8.24 (s),8.62 (s)	1.30 ± 0.41	1.16 ± 0.17	1.07 ± 0.16	0.69 ± 0.22	0.61 ± 0.09	0.56 ± 0.08
54	ADP	4.2 (m), 4.37 (m), 4.57 (dd), 4.74 (dd), 6.13 (dd), 8.27 (s),8.58 (s)	0.69 ± 0.08	0.37 ± 0.02	0.41 ± 0.05	0.36 ± 0.04	0.19 ± 0.01	0.22 ± 0.03
55	ATP	4.2 (m), 4.4 (m), 4.57 (dd), 4.74 (dd), 6.15 (d), 8.23 (s),8.38 (s)	0.39 ± 0.18	0.24 ± 0.04	0.56 ± 0.07	0.21 ± 0.10	0.13 ± 0.02	0.29 ± 0.04
56	GTP	4.24 (m), 4.35 (m), 4.55 (m), 4.74 (dd), 5.94 (d), 8.12 (s)	0.64 ± 0.11	0.61 ± 0.15	0.41 ± 0.06	0.34 ± 0.06	0.32 ± 0.08	0.22 ± 0.03
57	Uracil	5.8 (d), 7.53 (d)	0.06 ± 0.01	0.14 ± 0.02	0.22 ± 0.06	0.03 ± 0.01	0.07 ± 0.01	0.11 ± 0.03
58	Fumarate	6.52 (s)	0.08 ± 0.02	0.08 ± 0.02	0.08 ± 0.02	0.04 ± 0.01	0.04 ± 0.01	0.04 ± 0.01
59	Niacinamide	7.6 (dd), 8.26 (m), 8.71 (m), 8.94 (d)	0.31 ± 0.09	0.34 ± 0.04	0.42 ± 0.05	0.16 ± 0.05	0.18 ± 0.02	0.22 ± 0.03
60	Oxypurinol	8.19 (s)	19.09 ± 2.98	10.28 ± 1.49	20.43 ± 0.90	10.05 ± 1.57	5.41 ± 0.78	10.75 ± 0.47
61	Hypoxanthine	8.19 (s), 8.21 (s)	0.50 ± 0.07	0.59 ± 0.20	0.68 ± 0.06	0.26 ± 0.04	0.31 ± 0.10	0.36 ± 0.03

s: singlet; d: doublet; t: triplet; q: quartet; m: multiplet; dd: doublet of doublet

Abbreviations: UDP-Glucuronate: Uridine Diphosphate Glucoronate; UDP-Galactose: Uridine Diphosphate Galactose; AMP: Adenosine Triphosphate; ADP: Adenosine Diphosphate; GTP: Guanosine-5'-Triphosphate

**Table 2:** Gamma radiation induced metabolic changes in spleen tissue extracts.

Key	Metabolites	Correlation coefficient	
		3 Gy	7.8 Gy
1	Leucine	0.867	0.92
2	2-Aminobutyrate	0.962	0.898
3	Valine	0.953	0.958
8	Threonine	0.877	0.870^*^
9	Lactate	0.973	0.985
11	Alanine	0.768^*^	0.937
12	Arginine	0.936	0.955
13	γ-Glutamylphenylalanine	0.448^*^	0.906
15	Glutathione	0.839	0.911
16	Malate	0.875	0.726^*^
18	2-Oxoglutarate	0.872	0.941
20	β-Alanine	0.563^*^	0.894
24	Tyramine	0.782^*^	0.945
26	Creatine	0.969	0.967
27	Creatinine	0.084^*^	0.956
28	Tyrosine	0.951	0.99
29	Phenylalanine	0.913	0.943
33	π-Methylhistidine	0.813	0.928
38	Taurine	0.949	0.922
40	myo-Inositol	0.95	0.961
41	Tryptophan	0.923	0.873^*^
44	Glycerol	0.871	0.936
45	Glycine	0.851	0.253^*^
54	3-Hydroxybutyrate	0.019*	−0.913
54	ADP	−0.914	−0.899
57	Uracil	0.939	0.937
60	Oxypurinol	−0.863	0.276*
