# Supplementary material for: 1H NMR Metabolomics Study of Spleen from C57BL/6 Mice Exposed to Gamma Radiation
Source: Metabolomics (Los Angel). Author manuscript; Available in PMC 2016 Mar 25. (PMC4807627; doi:10.4172/2153-0769.1000165)
Supplement: Suppl data [file NIHMS762231-supplement-Suppl_data.docx]

**Supporting information**

**^1^H NMR Metabolomics Study of Spleen from**

**C57BL/6 Mice Exposed to Gamma Radiation**

Xiongjie Xiao ^1, 2^, Mary Hu^1^, Maili Liu^2^, Jian Zhi Hu^1*^

1. Pacific Northwest National Laboratory, Richland, WA 99352, USA
2. Key Laboratory of Magnetic Resonance in Biological Systems, State Key Laboratory of Magnetic Resonance and Atomic and Molecular Physics, Wuhan Centre for Magnetic Resonance, Wuhan Institute of Physics and Mathematics, the Chinese Academy of Sciences, Wuhan, 430071, PR China.

* To whom correspondence should be addressed:

Jian Zhi Hu; Email: [Jianzhi.Hu@pnnl.gov](mailto:Jianzhi.Hu@pnnl.gov); Phone: (509) 371-6544; Fax: (509) 371-6546


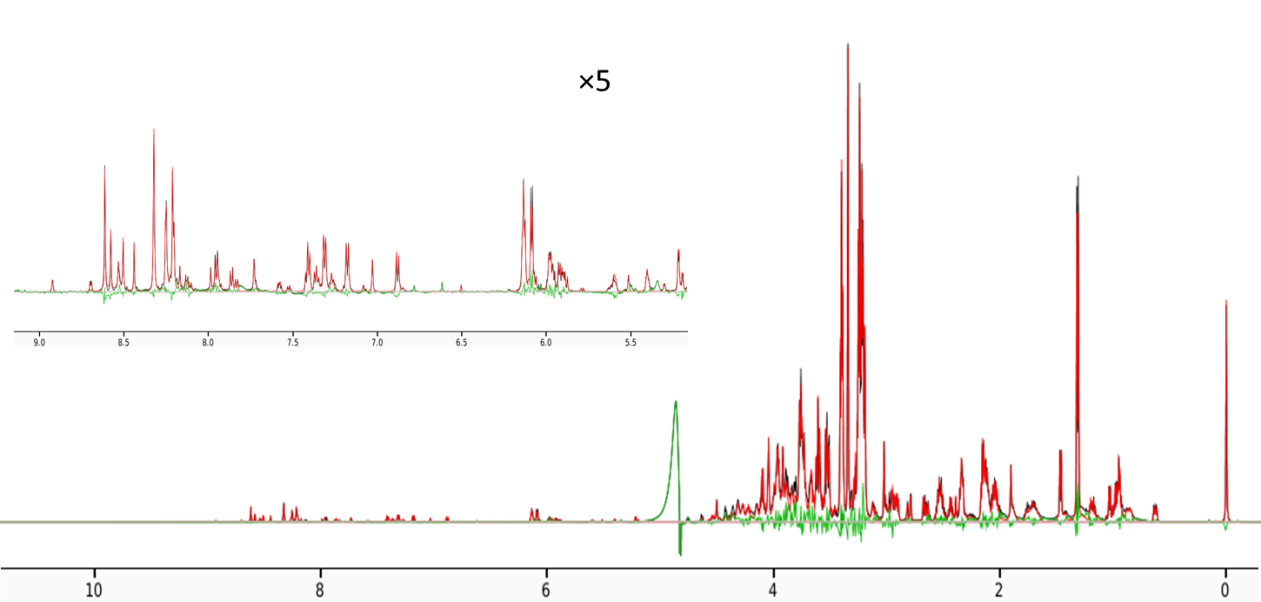


**Fig.S1**

Spectral deconvolution on a representative ^1^H NMR spectrum using Chenomx. The low field regions were vertically expanded 5 times compared with the high field regions of the spectrum. Black line is the experimental spectrum while red line shows the fitted spectrum. Green line indicates the fitting error.

**Table S1**

Solvent-Tissue ratio for extraction

| tissue | MeOH | H2O | CHCl3 | CHCl3 | H2O |
| --- | --- | --- | --- | --- | --- |
|  | (ml) | (μl) | (ml) | (ml) | (μl) |
| 1g | 4 | 850 | 2 | 2 | 2 |
| 0.5g | 2 | 425 | 1 | 1 | 1 |
| 0.25g | 1 | 213 | 0.5 | 0.5 | 0.5 |
| 125mg | 0.5 | 106 | 0.25 | 0.25 | 0.25 |
| 62.5mg | 0.25 | 53 | 0.125 | 0.125 | 0.125 |
| 31.3mg | 0.25 | 53 | 0.125 | 0.125 | 0.125 |
| 10.0mg | 0.25 | 53 | 0.125 | 0.125 | 0.125 |

**Table S2**

OPLS correlation coefficients of all metabolite concentrations with normalization to unit weight

| Key | Metabolites | Correlation coefficient | |
| --- | --- | --- | --- |
|  |  | 3Gy | 7.8Gy |
| 1 | Leucine | 0.867 | 0.920 |
| 2 | 2-Aminobutyrate | 0.962 | 0.898 |
| 3 | Valine | 0.953 | 0.958 |
| 4 | Isobutyrate | -0.623 | 0.047 |
| 5 | 3-Hydroxyisobutyrate | -0.722 | 0.364 |
| 6 | 3-Hydroxybutyrate | 0.019 | -0.913 |
| 7 | Fucose | 0.547 | 0.614 |
| 8 | Threonine | 0.877 | 0.870 |
| 9 | Lactate | 0.973 | 0.985 |
| 10 | Lysine | 0.759 | 0.781 |
| 11 | Alanine | 0.768 | 0.937 |
| 12 | Arginine | 0.936 | 0.955 |
| 13 | γ-Glutamylphenylalanine | 0.448 | 0.906 |
| 14 | Glutamate | 0.124 | -0.422 |
| 15 | Glutathione | 0.839 | 0.911 |
| 16 | Malate | 0.875 | 0.726 |
| 17 | Succinate | -0.116 | -0.370 |
| 18 | 2-Oxoglutarate | 0.872 | 0.941 |
| 19 | Isocitrate | -0.169 | 0.730 |
| 20 | β-Alanine | 0.563 | 0.894 |
| 21 | Citrate | 0.504 | 0.514 |
| 22 | Aspartate | 0.283 | 0.363 |
| 23 | Trimethylamine | 0.133 | 0.122 |
| 24 | Tyramine | 0.782 | 0.945 |
| 25 | Creatine phosphate | 0.302 | 0.844 |
| 26 | Creatine | 0.969 | 0.967 |
| 27 | Creatinine | 0.084 | 0.956 |
| 28 | Tyrosine | 0.951 | 0.990 |
| 29 | Phenylalanine | 0.913 | 0.943 |
| 30 | Histidine | -0.480 | 0.603 |
| 31 | Ethanolamine | -0.301 | 0.140 |
| 32 | Choline | -0.792 | 0.378 |
| 33 | π-Methylhistidine | 0.813 | 0.928 |
| 34 | O-Phosphoethanolamine | -0.628 | -0.862 |
| 35 | Glucose | 0.015 | 0.792 |
| 36 | Trimethylamine N-oxide | -0.622 | 0.110 |
| 37 | sn-Glycero-3-phosphocholine | 0.811 | 0.786 |
| 38 | Taurine | 0.949 | 0.922 |
| 39 | Betaine | -0.681 | -0.691 |
| 40 | myo-Inositol | 0.950 | 0.961 |
| 41 | Tryptophan | 0.923 | 0.873 |
| 42 | UDP-glucose | -0.766 | -0.800 |
| 43 | UDP-glucuronate | -0.602 | -0.859 |
| 44 | Glycerol | 0.871 | 0.936 |
| 45 | Glycine | 0.851 | 0.253 |
| 46 | UDP-galactose | 0.301 | 0.386 |
| 47 | Ascorbate | -0.463 | -0.648 |
| 48 | Uridine | 0.516 | -0.053 |
| 49 | Cytidine | -0.568 | -0.529 |
| 50 | Adenosine | -0.151 | -0.726 |
| 51 | Inosine | -0.543 | -0.529 |
| 52 | Serine | 0.283 | 0.744 |
| 53 | AMP | -0.165 | -0.329 |
| 54 | ADP | -0.914 | -0.899 |
| 55 | ATP | -0.540 | 0.484 |
| 56 | GTP | -0.121 | -0.782 |
| 57 | Uracil | 0.939 | 0.937 |
| 58 | Fumarate | 0.110 | -0.118 |
| 59 | Niacinamide | 0.216 | 0.602 |
| 60 | Oxypurinol | -0.863 | 0.276 |
| 61 | Hypoxanthine | 0.391 | 0.844 |

**Table S3**

3 Gy radiation metabolites normalization to unit weight and constant sum

| key | Metabolites | Correlation coefficients | |
| --- | --- | --- | --- |
|  |  | Unit weight | Constant sum |
| 1 | Leucine | 0.867 | 0.857 |
| 2 | 2-Aminobutyrate | 0.962 | 0.867 |
| 3 | Valine | 0.953 | 0.956 |
| 8 | Threonine | 0.877 | 0.854 |
| 9 | Lactate | 0.973 | 0.949 |
| 12 | Arginine | 0.936 | 0.894 |
| 15 | Glutathione | 0.839 | 0.710* |
| 16 | Malate | 0.875 | 0.694* |
| 18 | 2-Oxoglutarate | 0.872 | 0.671* |
| 26 | Creatine | 0.969 | 0.957 |
| 28 | Tyrosine | 0.951 | 0.928 |
| 29 | Phenylalanine | 0.912 | 0.936 |
| 33 | π-Methylhistidine | 0.813 | 0.729* |
| 34 | O-Phosphoethanolamine | -0.628* | -0.881 |
| 37 | sn-Glycero-3-phosphocholine | 0.810* | 0.832 |
| 38 | Taurine | 0.949 | 0.483* |
| 40 | myo-Inositol | 0.949 | 0.965 |
| 41 | Tryptophan | 0.923 | 0.863 |
| 44 | Glycerol | 0.871 | 0.778* |
| 45 | Glycine | 0.851 | 0.745* |
| 54 | ADP | -0.914 | -0.91 |
| 57 | Uracil | 0.939 | 0.939 |
| 60 | Oxypurinol | -0.863 | -0.878 |

* indicates metabolites that are not statistically and significantly important.

**Table S4**

7.8 Gy radiation metabolites normalization to unit weight and constant sum

| Key | Metabolites | Correlation coefficients | |
| --- | --- | --- | --- |
|  |  | Unit weight | Constant sum |
| 1 | Leucine | 0.919 | 0.842* |
| 2 | 2-Aminobutyrate | 0.898 | 0.670* |
| 3 | Valine | 0.958 | 0.92 |
| 6 | 3-Hydroxybutyrate | -0.913 | -0.951 |
| 9 | Lactate | 0.985 | 0.96 |
| 11 | Alanine | 0.937 | 0.890* |
| 12 | Arginine | 0.955 | 0.844* |
| 13 | γ-Glutamylphenylalanine | 0.906 | 0.624* |
| 15 | Glutathione | 0.911 | 0.806* |
| 18 | 2-Oxoglutarate | 0.941 | 0.861* |
| 20 | β-Alanine | 0.894 | 0.599* |
| 24 | Tyramine | 0.945 | 0.906 |
| 26 | Creatine | 0.966 | 0.964 |
| 27 | Creatinine | 0.956 | 0.898 |
| 28 | Tyrosine | 0.989 | 0.964 |
| 29 | Phenylalanine | 0.943 | 0.887 |
| 33 | π-Methylhistidine | 0.928 | 0.846* |
| 34 | O-Phosphoethanolamine | -0.862* | -0.958 |
| 38 | Taurine | 0.922 | 0.665* |
| 40 | myo-Inositol | 0.961 | 0.976 |
| 42 | UDP-glucose | -0.800* | -0.919 |
| 43 | UDP-glucuronate | -0.859* | -0.891 |
| 44 | Glycerol | 0.936 | 0.921 |
| 54 | ADP | -0.899 | -0.894 |
| 57 | Uracil | 0.937 | 0.889 |

* indicates metabolites that are not statistically and significantly important.

**Table S5**

Metabolites changed between 3 Gy and 7.8 Gy radiation groups

| Key | Metabolites | Correlation coefficients | |
| --- | --- | --- | --- |
|  |  | Unit weight | Constant sum |
| 3 | Valine | 0.927 | 0.798* |
| 5 | 3-Hydroxybutyrate | -0.957 | -0.909 |
| 6 | 3-Hydroxyisobutyrate | 0.893 | 0.766* |
| 9 | Lactate | 0.917 | 0.603* |
| 14 | Glutamate | -0.762* | -0.902 |
| 19 | Isocitrate | 0.918 | 0.655* |
| 24 | Tyramine | 0.937 | 0.864* |
| 25 | Creatine phosphate | 0.896 | 0.757* |
| 27 | Creatinine | 0.969 | 0.958 |
| 28 | Tyrosine | 0.937 | 0.770* |
| 32 | Choline | 0.946 | 0.863* |
| 34 | O-Phosphoethanolamine | -0.643* | -0.889 |
| 40 | myo-Inositol | 0.825* | 0.886 |
| 52 | Serine | 0.880 | 0.303* |
| 55 | ATP | 0.951 | 0.900 |
| 60 | Oxypurinol | 0.978 | 0.955 |

* indicates metabolites that are not statistically and significantly important.


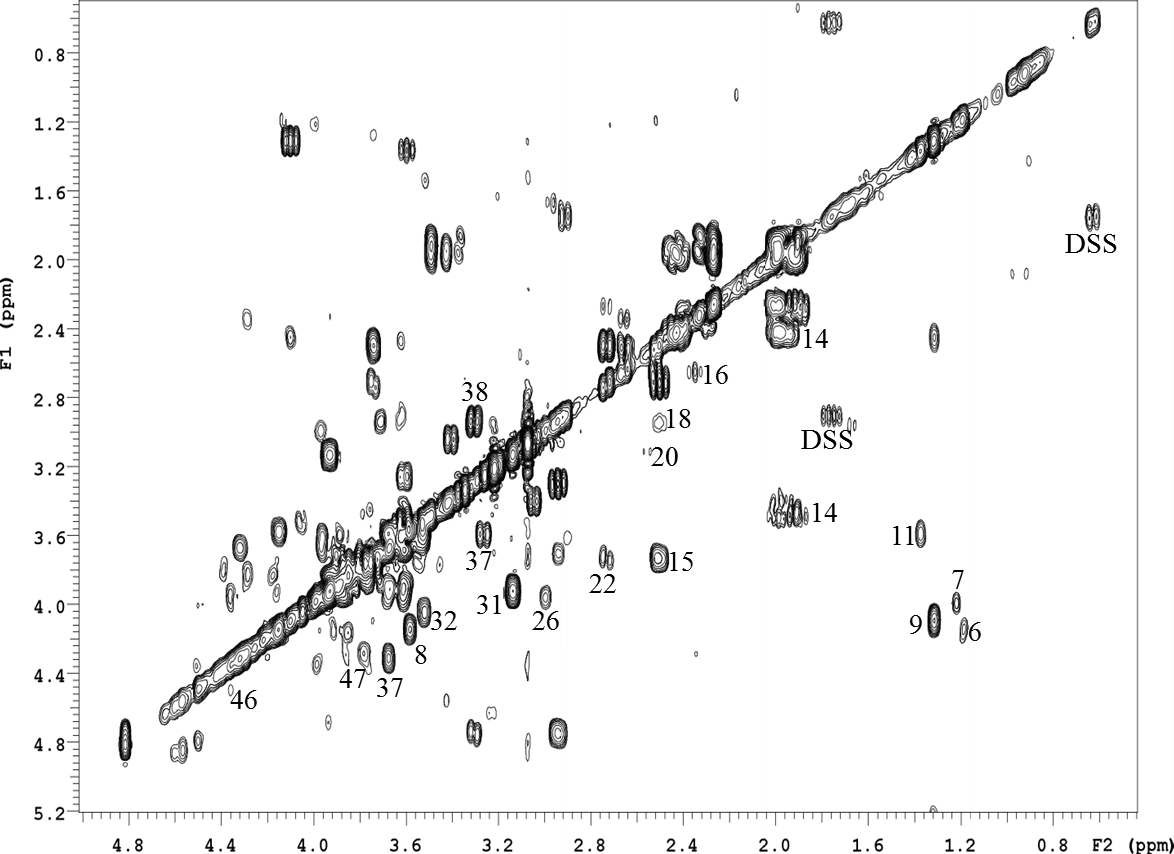


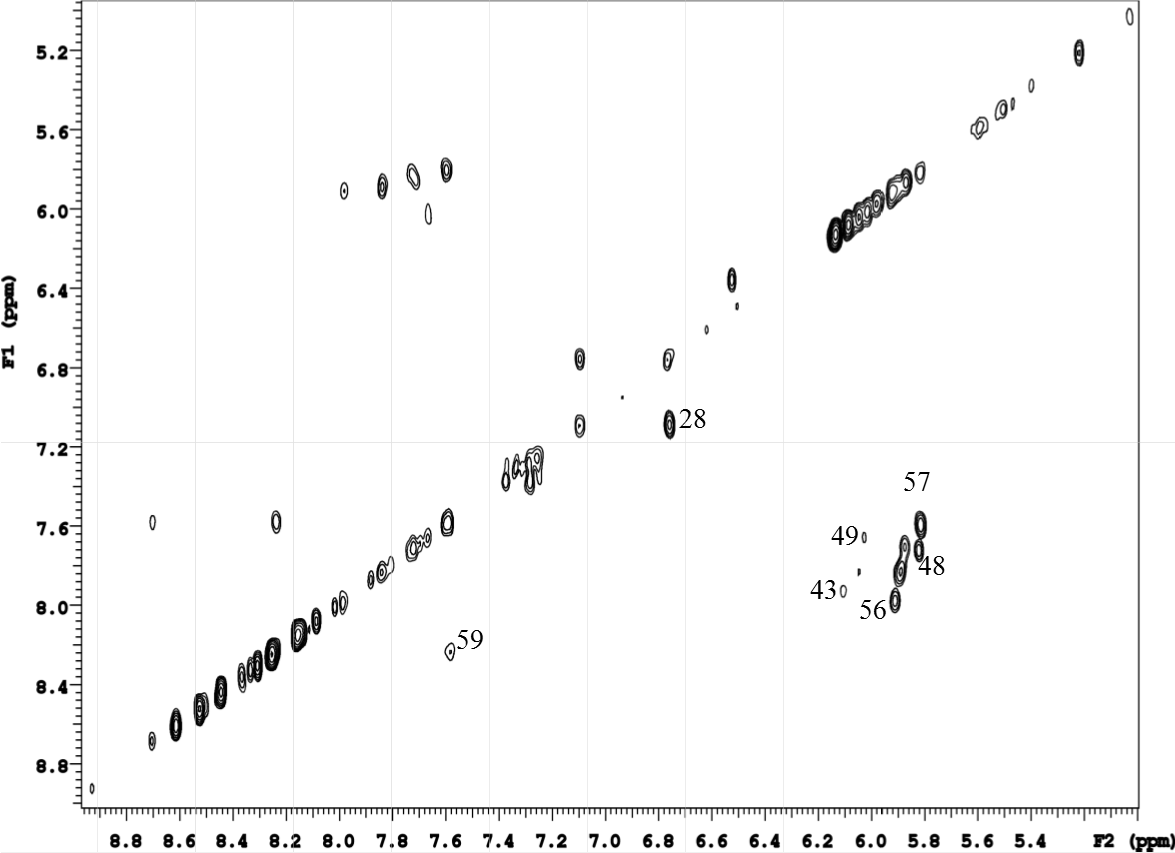


**Fig.S2**

600 MHz ^1^H-^1^H COSY NMR spectrum of extracts


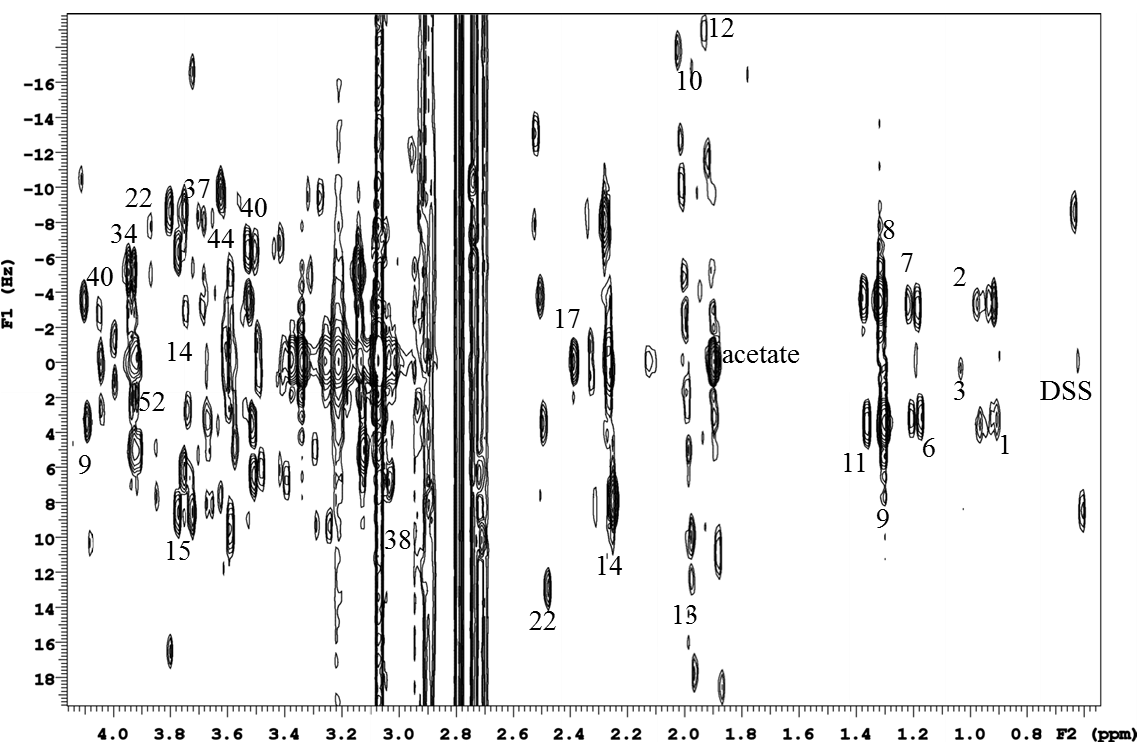

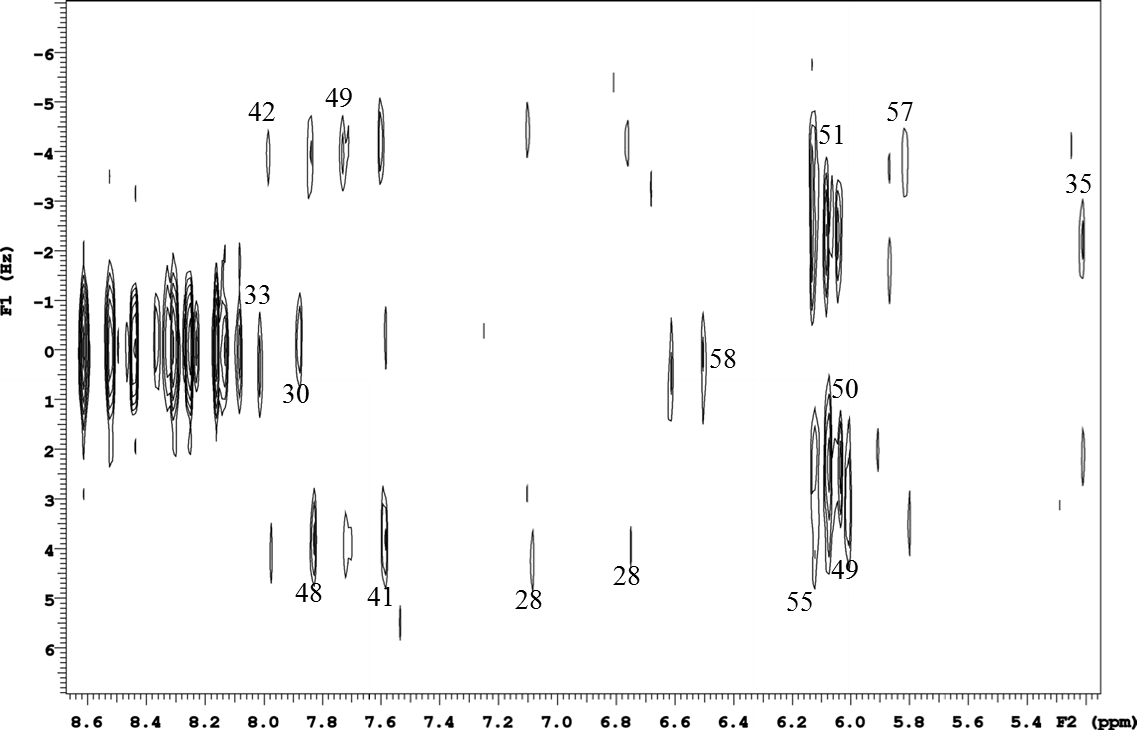


**Fig.S3**

600 MHz 2D ^1^H-^1^H homonuclear J-resolved of extracts

1, leucine; 2, 2-aminobutyrate; 3, valine; 6, 3-hydroxybutyrate; 7, fucose; 8, threonine; 9, lactate; 11, alanine; 12, arginine; 13, γ-glutamylphenylalanine; 14, glutamate; 15, glutathione; 16, malate; 17, succinate; 18, 2-oxoglutarate; 20, β-alanine; 22, aspartate; 26, creatine; 28, tyrosine; 30, histidine; 31, ethanolamine; 32, choline; 33, π-methylhistidine; 34, o-phosphoethanolamine; 35, glucose; 37, sn-glycero-3-phosphocholine; 38, taurine; 40, myo-inositol; 41, tryptophan; 42, UDP-glucose; 43, UDP-glucuronate; 44, glycerol; 46, UDP-galactose; 47, ascorbate; 48, uridine; 49, cytidine; 50, adenosine; 51, inosine; 52, serine; 55, ATP; 56, GTP; 57, uracil; 58, fumarate; 59, niacinamide.

**Table S6**

OPLS model statistical parameters based on spectral binning and spectral deconvolution

| samples | metadata | normalization | R^2^X | Q^2^ | p |
| --- | --- | --- | --- | --- | --- |
| 3Gy Vs. Control | absolute bin | unit weight | 0.766 | 0.829 | 0.095 |
|  | absolute concentration |  | 0.443 | 0.945 | 5.05×10^-7^ |
|  | relative bin | constant sum | 0.675 | 0.864 | 0.02 |
|  | relative concentration |  | 0.520 | 0.968 | 5.07×10^-6^ |
| 7.8Gy Vs. Control | absolute bin | unit weight | 0.476 | 0.855 | 4.6×10^-3^ |
|  | absolute concentration |  | 0.545 | 0.981 | 1.96×10^-8^ |
|  | relative bin | constant sum | 0.459 | 0.925 | 4.8×10^-4^ |
|  | relative concentration |  | 0.571 | 0.997 | 5.64×10^-6^ |

**Materials and Methods**

Animal Experiments and Sample Preparation

Groups of mice were exposed to radiation doses of 0 Gy, 3.0 Gy and 7.8 Gy. The reasons for selecting these radiation doses are explained below. (i) Metabolomics has been successfully utilized for assessing potential biomarkers in urine [1], plasma [2] and serum [3] from mice exposed to 3 Gy gamma radiation, and interesting results have been obtained. (ii) The lethal dose for Balb/c mice is about 7.8 Gy [4], and in the case of an atomic bomb attack, survivors may expose to high dose radiation of 7.8 Gy or even higher.

It has been reported [1] that 24h after exposure to high dose gamma radiation of 3 and 8 Gy, metabolites were found changed in urine. However, 3 days after exposed to the lethal dose gamma radiation, the level of metabolites in urine samples of nonhuman primates has some outliers when compared with controls [5], indicating significant biological variations. In the present study, our focus is on studying the metabolite changes in spleen tissue and we would like to investigate at a time point close to 3 day post exposure, and therefore 4 days post exposure was selected in the present study.”

1. Tyburski, J.B., et al., *Radiation metabolomics. 1. Identification of minimally invasive urine biomarkers for gamma-radiation exposure in mice.* Radiation Research, 2008. **170**(1): p. 1-14.

2. Wang, C., J. Yang, and J.H. Nie, *Plasma phospholipid metabolic profiling and biomarkers of rats following radiation exposure based on liquid chromatography-mass spectrometry technique.* Biomedical Chromatography, 2009. **23**(10): p. 1079-1085.

3. Khan, A.R., et al., *Nuclear magnetic resonance spectroscopy-based metabonomic investigation of biochemical effects in serum of gamma-irradiated mice.* International Journal of Radiation Biology, 2011. **87**(1): p. 91-97.

4. Mortazavi, S.M.J., et al., *Increased Radioresistance to Lethal Doses of Gamma Rays in Mice and Rats after Exposure to Microwave Radiation Emitted by a Gsm Mobile Phone Simulator.* Dose-Response, 2013. **11**(2): p. 281-292.

5. Johnson, C.H., et al., *Radiation Metabolomics. 5. Identification of Urinary Biomarkers of Ionizing Radiation Exposure in Nonhuman Primates by Mass Spectrometry-Based Metabolomics.* Radiation Research, 2012. **178**(4): p. 328-340.
